# Effectiveness of Face Mask or Respirator Use in Indoor Public Settings for Prevention of SARS-CoV-2 Infection — California, February–December 2021

**DOI:** 10.15585/mmwr.mm7106e1

**Published:** 2022-02-11

**Authors:** Kristin L. Andrejko, Jake M. Pry, Jennifer F. Myers, Nozomi Fukui, Jennifer L. DeGuzman, John Openshaw, James P. Watt, Joseph A. Lewnard, Seema Jain, California COVID-19 Case-Control Study Team, California COVID-19 Case-Control Study Team, Yasmine Abdulrahim, Camilla M. Barbaduomo, Miriam I. Bermejo, Julia Cheunkarndee, Adrian F. Cornejo, Savannah Corredor, Najla Dabbagh, Zheng N. Dong, Ashly Dyke, Anna T. Fang, Diana Felipe, Paulina M. Frost, Timothy Ho, Mahsa H. Javadi, Amandeep Kaur, Amanda Lam, Sophia S. Li, Monique Miller, Jessica Ni, Hyemin Park, Diana J. Poindexter, Helia Samani, Shrey Saretha, Maya Spencer, Michelle M. Spinosa, Vivian H. Tran, Nikolina Walas, Christine Wan, Erin Xavier

**Affiliations:** ^1^Division of Epidemiology and Biostatistics, School of Public Health, University of California, Berkeley, California; ^2^California Department of Public Health; ^3^Division of Infectious Diseases & Vaccinology, School of Public Health, University of California, Berkeley, California; ^4^Center for Computational Biology, College of Engineering, University of California, Berkeley, California.; California Department of Public Health; California Department of Public Health; California Department of Public Health; California Department of Public Health; California Department of Public Health; California Department of Public Health; California Department of Public Health; California Department of Public Health; California Department of Public Health; California Department of Public Health; California Department of Public Health; California Department of Public Health; California Department of Public Health; California Department of Public Health; California Department of Public Health; California Department of Public Health; California Department of Public Health; California Department of Public Health; California Department of Public Health; California Department of Public Health; California Department of Public Health; California Department of Public Health; California Department of Public Health; California Department of Public Health; California Department of Public Health; California Department of Public Health; California Department of Public Health; California Department of Public Health; California Department of Public Health

The use of face masks or respirators (N95/KN95) is recommended to reduce transmission of SARS-CoV-2, the virus that causes COVID-19 (*1*). Well-fitting face masks and respirators effectively filter virus-sized particles in laboratory conditions (*2*,*3*), though few studies have assessed their real-world effectiveness in preventing acquisition of SARS-CoV-2 infection (*4*). A test-negative design case-control study enrolled randomly selected California residents who had received a test result for SARS-CoV-2 during February 18–December 1, 2021. Face mask or respirator use was assessed among 652 case-participants (residents who had received positive test results for SARS-CoV-2) and 1,176 matched control-participants (residents who had received negative test results for SARS-CoV-2) who self-reported being in indoor public settings during the 2 weeks preceding testing and who reported no known contact with anyone with confirmed or suspected SARS-CoV-2 infection during this time. Always using a face mask or respirator in indoor public settings was associated with lower adjusted odds of a positive test result compared with never wearing a face mask or respirator in these settings (adjusted odds ratio [aOR] = 0.44; 95% CI = 0.24–0.82). Among 534 participants who specified the type of face covering they typically used, wearing N95/KN95 respirators (aOR = 0.17; 95% CI = 0.05–0.64) or surgical masks (aOR = 0.34; 95% CI = 0.13–0.90) was associated with significantly lower adjusted odds of a positive test result compared with not wearing any face mask or respirator. These findings reinforce that in addition to being up to date with recommended COVID-19 vaccinations, consistently wearing a face mask or respirator in indoor public settings reduces the risk of acquiring SARS-CoV-2 infection. Using a respirator offers the highest level of personal protection against acquiring infection, although it is most important to wear a mask or respirator that is comfortable and can be used consistently.

This study used a test-negative case-control design, enrolling persons who received a positive (case-participants) or negative (control-participants) SARS-CoV-2 test result, from among all California residents, without age restriction, who received a molecular test result for SARS-CoV-2 during February 18–December 1, 2021 (*5*). Potential case-participants were randomly selected from among all persons who received a positive test result during the previous 48 hours and were invited to participate by telephone. For each enrolled case-participant, interviewers enrolled one control-participant matched by age group, sex, and state region; thus, interviewers were not blinded to participants’ SARS-CoV-2 infection status. Participants who self-reported having received a previous positive test result (molecular, antigen, or serologic) or clinical diagnosis of COVID-19 were not eligible to participate. During February 18–December 1, 2021, a total of 1,528 case-participants and 1,511 control-participants were enrolled in the study among attempted calls placed to 11,387 case- and 17,051 control-participants (response rates were 13.4% and 8.9%, respectively).

After obtaining informed consent from participants, interviewers administered a telephone questionnaire in English or Spanish. All participants were asked to indicate whether they had been in indoor public settings (e.g., retail stores, restaurants or bars, recreational facilities, public transit, salons, movie theaters, worship services, schools, or museums) in the 14 days preceding testing and whether they wore a face mask or respirator all, most, some, or none of the time in those settings. Interviewers recorded participants’ responses regarding COVID-19 vaccination status, sociodemographic characteristics, and history of exposure to anyone known or suspected to have been infected with SARS-CoV-2 in the 14 days before participants were tested. Participants enrolled during September 9–December 1, 2021, (534) were also asked to indicate the type of face covering typically worn (N95/KN95 respirator, surgical mask, or cloth mask) in indoor public settings.

The primary analysis compared self-reported face mask or respirator use in indoor public settings 14 days before SARS-CoV-2 testing between case- (652) and control- (1,176) participants. Secondary analyses accounted for consistency of face mask or respirator use all, most, some, or none of the time. To understand the effects of masking on community transmission, the analysis included the subset of participants who, during the 14 days before they were tested, reported visiting indoor public settings and who reported no known exposure to persons known or suspected to have been infected with SARS-CoV-2. An additional analysis assessed differences in protection against SARS-CoV-2 infection by the type of face covering worn, and was limited to a subset of participants enrolled after September 9, 2021, who were asked to indicate the type of face covering they typically wore; participants who indicated typically wearing multiple different mask types were categorized as wearing either a cloth mask (if they reported cloth mask use) or a surgical mask (if they did not report cloth mask use). Adjusted odds ratios comparing history of mask-wearing among case- and control-participants were calculated using conditional logistic regression. Match strata were defined by participants’ week of SARS-CoV-2 testing and by county-level SARS-CoV-2 risk tiers as defined under California’s Blueprint for a Safer Economy reopening scheme.^†^ Adjusted models accounted for self-reported COVID-19 vaccination status (fully vaccinated with ≥2 doses of BNT162b2 [Pfizer-BioNTech] or mRNA-1273 [Moderna] or 1 dose of Ad.26.COV2.S [Janssen (Johnson & Johnson)] vaccine >14 days before testing versus zero doses), household income, race/ethnicity, age, sex, state region, and county population density. Statistical significance was defined by two-sided Wald tests with p-values <0.05. All analyses were conducted using R software (version 3.6.1; R Foundation). This activity was approved as public health surveillance by the State of California Health and Human Services Agency Committee for the Protection of Human Subjects.

A total of 652 case- and 1,176 control-participants were enrolled in the study equally across nine multi-county regions in California (Table 1). The majority of participants (43.2%) identified as non-Hispanic White; 28.2% of participants identified as Hispanic (any race). A higher proportion of case-participants (78.4%) was unvaccinated compared with control-participants (57.5%). Overall, 44 (6.7%) case-participants and 42 (3.6%) control-participants reported never wearing a face mask or respirator in indoor public settings (Table 2), and 393 (60.3%) case-participants and 819 (69.6%) control-participants reported always wearing a face mask or respirator in indoor public settings. Any face mask or respirator use in indoor public settings was associated with significantly lower odds of a positive test result compared with never using a face mask or respirator (aOR = 0.51; 95% CI = 0.29­–0.93). Always using a face mask or respirator in indoor public settings was associated with lower adjusted odds of a positive test result compared with never wearing a face mask or respirator (aOR = 0.44; 95% CI = 0.24–0.82); however, adjusted odds of a positive test result suggested stepwise reductions in protection among participants who reported wearing a face mask or respirator most of the time (aOR = 0.55; 95% CI = 0.29–1.05) or some of the time (aOR = 0.71; 95% CI = 0.35–1.46) compared with participants who reported never wearing a face mask or respirator.

**TABLE 1:** Characteristics of case- and control-participants included in analysis of the effectiveness of mask use in indoor public settings, by SARS-CoV-2 test result — California,* February–December 2021

Characteristic	No. (%)	
	Case-participants (SARS-CoV-2–positive)	Control-participants (SARS-CoV-2–negative)
	N = 652	N = 1,176
**Age group, yrs**		
0–6	8 (1.2)	43 (3.7)
7–12	15 (2.3)	49 (4.2)
13–17	25 (3.8)	57 (4.8)
18–29	210 (32.2)	359 (30.5)
30–49	237 (36.3)	409 (34.8)
50–64	109 (16.7)	180 (15.3)
≥65	48 (7.4)	79 (6.7)
**Sex**		
Male	321 (49.2)	581 (49.4)
Female	331 (50.8)	595 (50.6)
**Annual household income**		
<$50,000	191 (29.3)	258 (21.9)
$50,000–$99,999	147 (22.5)	254 (21.6)
$100,000–$150,000	60 (9.2)	171 (14.5)
>$150,000	77 (11.8)	197 (16.8)
Refused	106 (16.3)	184 (15.6)
Not sure	71 (10.9)	112 (9.5)
**State region^†^**		
San Francisco Bay Area	79 (12.1)	147 (12.5)
Greater Los Angeles Area	77 (11.8)	130 (11.1)
Greater Sacramento Area	53 (8.1)	131 (11.1)
San Diego and southern border	73 (11.2)	142 (12.1)
Central Coast	87 (13.3)	132 (11.2)
Northern Sacramento Valley	69 (10.6)	134 (11.4)
San Joaquin Valley	79 (12.1)	130 (11.1)
Northwestern California	78 (12.0)	113 (9.6)
Sierras	57 (8.7)	117 (9.9)
**Race/Ethnicity**		
White, non-Hispanic	292 (44.8)	506 (43.0)
Black, non-Hispanic	39 (6.0)	42 (3.6)
Hispanic (any race)	201 (30.8)	315 (26.8)
Asian, non-Hispanic	56 (8.6)	134 (11.4)
American Indian or Alaska Native, non-Hispanic	9 (1.4)	10 (0.9)
Native Hawaiian or Other Pacific Islander, non-Hispanic	2 (0.3)	12 (1.0)
More than one race	40 (6.1)	131 (11.1)
Refused	13 (2.0)	26 (2.2)
**COVID-19 vaccination status** ^§^		
Unvaccinated or incompletely vaccinated	511 (78.4)	676 (57.5)
Fully vaccinated	115 (17.6)	377 (32.1)
Unknown	26 (4.0)	123 (10.5)
**Reopening tier in California** ^¶^		
Tier 1 (most restrictive)	125 (19.2)	237 (20.2)
Tier 2	152 (23.3)	255 (21.7)
Tier 3	119 (18.3)	272 (23.1)
Tier 4 (least restrictive)	18 (2.8)	32 (2.7)
After June 15, 2021	238 (36.5)	380 (32.3)
Reasons for SARS-CoV-2 testing**		
Experiencing symptoms	508 (77.9)	196 (16.7)
Testing required for medical procedure	40 (6.1)	199 (16.9)
Routine screening through work or school	71 (10.9)	507 (43.1)
Pre-travel test	33 (5.1)	120 (10.2)
Just wanted to see if I was infected	65 (10.0)	172 (14.6)
Test required for admission to an event or gathering	3 (0.5)	21 (1.8)

* A random sample of California residents with a molecular SARS-CoV-2 test result was invited to participate in a telephone-based survey to document frequency of face mask or respirator use and type of face mask or respirator typically worn in indoor public settings 2 weeks before testing. For each enrolled case-participant (person with a positive SARS-CoV-2 test result), interviewers attempted to enroll one control-participant (person with a negative SARS-CoV-2 test result) whose test result was posted to the reportable disease registry during the 48 hours preceding the call and matched the case-participant by age group, sex, and state region. Among 1,947 case- and control-participants who visited indoor public settings and did not report a known or suspected exposure to SARS-CoV-2 in the 14 days before getting a SARS-CoV-2 test, 119 (6.1%) participants were unable to report face mask use and were excluded from analysis. Parents or guardians served as proxy respondents and answered questions throughout the telephone survey on behalf of children aged <13 years.

**^†^** California counties were divided into nine geographic regions. Counties included in each geographic region are listed online in Table S1. https://academic.oup.com/cid/advance-article/doi/10.1093/cid/ciab640/6324500#supplementary-data

^§^ Vaccination status was defined using self-reported dates and manufacturers of doses received. Participants were asked to reference their COVID-19 vaccination card while providing vaccination history. Participants who could not provide a complete vaccination history (dates of doses received and manufacturers) were coded as unknown. Full vaccination was defined as receipt of 2 doses of BNT162b2 [Pfizer-BioNTech] or mRNA-1273 [Moderna], or receipt of 1 dose of Ad.26.COV2.S (Janssen [Johnson & Johnson]) >14 days before SARS-CoV-2 testing. Of the 492 fully vaccinated participants, 22 (4.5%) had received a booster dose at the time of enrollment. All other participants were considered unvaccinated or incompletely vaccinated.

^¶^ Reopening tiers in California were determined by the Blueprint for a Safer Economy the State of California implemented during February 24 to June 15, 2021. This was a tiered system of public health restrictions tied to county-level positive test results and incidence. On June 15, 2021, California retired the tiered reopening system and removed most restrictions on public gatherings, while some counties maintained guidelines for guests and workers to show proof of vaccination or a negative test result to gather in certain types of venues and workplaces. The tier of a given participant was determined by using the date that occurred 14 days before the SARS-CoV-2 specimen collection date recorded for each participant in the California Reportable Disease Registry.

** Case- and control-participants were asked to indicate their reasons for seeking a SARS-CoV-2 test as a free-text response. Trained interviewers (N = 29) recategorized the free-text response into the categories listed in the table. Interviewers were trained to ask probing questions if the free-text response could not be categorized into the reasons listed above. Probing questions and coding decisions may slightly vary by interviewer. Reasons for testing might sum to numbers larger than the total number of case-participants or control-participants because participants could indicate more than one reason for seeking a SARS-CoV-2 test.

**TABLE 2:** Face mask or respirator use in indoor public settings among persons with positive and negative SARS-CoV-2 test results — California, February–December 2021

Mask type and use*	SARS-CoV-2 infection status, no. (%)		Odds ratio (95% CI)	
	Positive (case-participant) N = 652	Negative (control-participant) N = 1,176	Unadjusted^†^ [p-value]	Adjusted^§^ [p-value]
None (Ref)	44 (6.7)	42 (3.6)	—	—
Any use**^†^**	608 (93.3)	1,134 (96.4)	0.57 (0.37–0.90) [0.02]	0.51 (0.29–0.93) [0.03]
Some of the time	62 (9.5)	76 (6.5)	0.81 (0.47–1.41) [0.49]	0.71 (0.35–1.46) [0.36]
Most of the time	153 (23.5)	239 (20.3)	0.64 (0.40–1.05) [0.08]	0.55 (0.29–1.05) [0.07]
All of the time	393 (60.3)	819 (69.6)	0.49 (0.31–0.78) [<0.01]	0.44 (0.24–0.82) [<0.01]

**Abbreviation:** Ref = referent group.

* Trained interviewers administered a structured telephone-based questionnaire and asked participants to indicate whether they attended indoor public spaces during the 2 weeks before seeking a SARS-CoV-2 test. Participants who indicated attending these settings were further asked to specify whether they typically wore a face mask or respirator all, most, some, or none of the time while in these settings.

**^†^** Conditional logistic regression models were used to estimate the unadjusted odds of mask use by type of face mask or respirator worn in indoor public settings during the 2 weeks before testing. Models included matching strata defined by (for the period before June 15, 2021) the reopening tier of California in the county of residence and the week of SARS-CoV-2 testing.

^§^ Conditional logistic regression models were used to estimate the odds of face mask or respirator use in indoor public settings during the 2 weeks before testing, adjusting for COVID-19 vaccination status, household income, race/ethnicity, age group, sex, state region, and county population density. All models included matching strata defined by (for the period before June 15, 2021) the reopening tier of California in the county of residence, and the week of SARS-CoV-2 testing. To understand the effects of masking in community settings, this analysis was restricted to a subset of persons who did not indicate a known or suspected exposure to a SARS-CoV-2 case within 14 days of seeking a SARS-CoV-2 test. Adjusted models used a complete case analysis (454 case-participants and 789 control-participants). A sensitivity analysis using multiple imputation of missing covariate values obtained results similar to those reported in the table: adjusted odds ratios were 0.54 (95% CI = 0.33–0.89) for any mask use, 0.44 (95% CI = 0.27–0.73) for mask use all of the time, 0.62 (95% CI = 0.37–1.04) for mask use most of the time, and 0.77 (95% CI = 0.43–1.40) for mask use some of the time. An additional sensitivity analysis was conducted with additional adjustment for the reasons for SARS-CoV-2 testing as listed in Table 1 (experiencing symptoms, testing required for medical procedure, routine screening through work or school, pre-travel test, just wanted to see if I was infected, test required for admission to an event or gathering). The adjusted odds ratio was 0.42 (95% CI = 0.20–0.89) for any mask use as compared to no mask use upon additional adjustment for testing indications.

Wearing an N95/KN95 respirator (aOR = 0.17; 95% CI = 0.05–0.64) or wearing a surgical mask (aOR = 0.34; 95% CI = 0.13­–0.90) was associated with lower adjusted odds of a positive test result compared with not wearing a mask (Table 3). Wearing a cloth mask (aOR = 0.44; 95% CI = 0.17–1.17) was associated with lower adjusted odds of a positive test compared with never wearing a face covering but was not statistically significant.

**TABLE 3:** Types of face mask or respirator worn in indoor public settings among persons with positive or negative SARS-CoV-2 test results — California, September–December 2021

Mask type*	SARS-CoV-2 infection status, no. (%)		Odds ratio (95% CI)	
	Positive (case-participant) N = 259	Negative (control-participant) N = 275	Unadjusted^†^ [p-value]	Adjusted^§^ [p-value]
None (Ref)	24 (9.3)	11 (4.0)	—	—
Cloth mask	112 (43.2)	104 (37.8)	0.50 (0.23–1.06) [0.07]	0.44 (0.17–1.17) [0.10]
Surgical mask	113 (43.6)	139 (50.5)	0.38 (0.18–0.81) [0.01]	0.34 (0.13–0.90) [0.03]
N95/KN95 respirator	10 (3.9)	21 (7.6)	0.22 (0.08–0.62) [<0.01]	0.17 (0.05–0.64) [<0.01]

**Abbreviation:** Ref = referent group.

* Trained interviewers administered a structured telephone-based questionnaire and asked participants enrolled after September 9, 2021, to identify the type of face covering typically worn in indoor public settings during the 2 weeks before seeking a SARS-CoV-2 test. Participants who indicated typically wearing multiple different mask types were categorized as wearing either a cloth mask (if they reported cloth mask use) or a surgical mask (if they didn’t report cloth mask use).

**^†^** Conditional logistic regression models were used to estimate the unadjusted odds of mask use by type of face mask or respirator worn in indoor public settings during the 2 weeks before testing. Models included matching strata defined by the week of SARS-CoV-2 testing.

^§^ This analysis was not restricted to persons with no self-reported known or suspected SARS-CoV-2 contact given that this secondary analysis was underpowered upon exclusion of these participants (N = 316) because adjusted models did not converge. Instead, models adjusted for history of known or suspected contact as a covariate. In a sensitivity analysis restricting to participants who did not report known or suspected contact (N = 316), conditional logistic regression models were used to estimate that the unadjusted odds ratios of face mask use by type of face mask with matching strata defined by the week of SARS-CoV-2 testing: 0.13 (95% CI = 0.03–0.61), 0.32 (95% CI = 0.12–0.89), and 0.36 (95% CI = 0.13–1.00) for N95/KN95 respirators, surgical masks, or cloth masks, respectively, relative to no face mask or respirator use.

## Discussion

During February–December 2021, using a face mask or respirator in indoor public settings was associated with lower odds of acquiring SARS-CoV-2 infection, with protection being highest among those who reported wearing a face mask or respirator all of the time. Although consistent use of any face mask or respirator indoors was protective, the adjusted odds of infection were lowest among persons who reported typically wearing an N95/KN95 respirator, followed by wearing a surgical mask. These data from real-world settings reinforce the importance of consistently wearing face masks or respirators to reduce the risk of acquisition of SARS-CoV-2 infection among the general public in indoor community settings.

These findings are consistent with existing research demonstrating that face masks or respirators effectively filter viruses in laboratory settings and with ecological studies showing reductions in SARS-CoV-2 incidence associated with community-level masking requirements (*6*,*7*). While this study evaluated the protective effects of mask or respirator use in reducing the risk the wearer acquires SARS-CoV-2 infection, a previous evaluation estimated the additional benefits of masking for source control, and found that wearing face masks or respirators in the context of exposure to a person with confirmed SARS-CoV-2 infection was associated with similar reductions in risk for infection (*8*). Strengths of the current study include use of a clinical endpoint of SARS-CoV-2 test result, and applicability to a general population sample.

The findings in this report are subject to at least eight limitations. First, this study did not account for other preventive behaviors that could influence risk for acquiring infection, including adherence to physical distancing recommendations. In addition, generalizability of this study is limited to persons seeking SARS-CoV-2 testing and who were willing to participate in a telephone interview, who might otherwise exercise other protective behaviors. Second, this analysis relied on an aggregate estimate of self-reported face mask or respirator use across, for some participants, multiple indoor public locations. However, the study was designed to minimize recall bias by enrolling both case- and control-participants within a 48-hour window of receiving a SARS-CoV-2 test result. Third, small strata limited the ability to differentiate between types of cloth masks or participants who wore different types of face masks in differing settings, and also resulted in wider CIs and statistical nonsignificance for some estimates that were suggestive of a protective effect. Fourth, estimates do not account for face mask or respirator fit or the correctness of face mask or respirator wearing; assessing the effectiveness of face mask or respirator use under real-world conditions is nonetheless important for developing policy. Fifth, data collection occurred before the expansion of the SARS-CoV-2 B.1.1.529 (Omicron) variant, which is more transmissible than earlier variants. Sixth, face mask or respirator use was self-reported, which could introduce social desirability bias. Seventh, small strata limited the ability to account for reasons for testing in the adjusted analysis, which may be correlated with face mask or respirator use. Finally, this analysis does not account for potential differences in the intensity of exposures, which could vary by duration, ventilation system, and activity in each of the various indoor public settings visited.

The findings of this report reinforce that in addition to being up to date with recommended COVID-19 vaccinations, consistently wearing face masks or respirators while in indoor public settings protects against the acquisition of SARS-CoV-2 infection (*9*,*10*). This highlights the importance of improving access to high-quality masks to ensure access is not a barrier to use. Using a respirator offers the highest level of protection from acquisition of SARS-CoV-2 infection, although it is most important to wear a well-fitting mask or respirator that is comfortable and can be used consistently.

**Summary**

**What is already known about this topic?**

Face masks or respirators (N95/KN95s) effectively filter virus-sized particles in laboratory settings. The real-world effectiveness of face coverings to prevent acquisition of SARS-CoV-2 infection has not been widely studied.

**What is added by this report?**

Consistent use of a face mask or respirator in indoor public settings was associated with lower odds of a positive SARS-CoV-2 test result (adjusted odds ratio = 0.44). Use of respirators with higher filtration capacity was associated with the most protection, compared with no mask use.

**What are the implications for public health practice?**

In addition to being up to date with recommended COVID-19 vaccinations, consistently wearing a comfortable, well-fitting face mask or respirator in indoor public settings protects against acquisition of SARS-CoV-2 infection; a respirator offers the best protection.

## Footnotes

* These authors contributed equally to this report.

†
https://www.cdph.ca.gov/Programs/CID/DCDC/Pages/COVID-19/COVID19CountyMonitoringOverview.aspx
